# 

**Published:** 1898-04

**Authors:** 


					﻿Vol. XVIII.
APRIL, 1898.
NO.V
THE
HOMEOPATHIC pHY£lClA]\I,
A Monthly Journal of Homoeopathic Materia Medica
and Clinical Medicine.
“ He it a freeman whom truth makes free, and all are slaves beside."
"Seek the 1 ruth: come whence it may, cost what it will."
•	EDITED AND PUBLISHED BY
WALTER M. JAMES, M. D.
PHILADELPHIA :
USTo. 1231 LOCUST STREET.
* Yearly Subscription« $2.60, invariably in advance. Single members9 cis.
Entered at the Philadelphia Post-Office as Second-Class Mail Matter.
				

